# Modeling Electro-Chemo-Mechanical Behaviors within the Dense BaZr_0.8_Y_0.2_O_3−*δ*_ Protonic-Ceramic Membrane in a Long Tubular Electrochemical Cell

**DOI:** 10.3390/membranes11060378

**Published:** 2021-05-22

**Authors:** Kasra Taghikhani, Alexis Dubois, John R. Berger, Sandrine Ricote, Huayang Zhu, Robert J. Kee

**Affiliations:** 1 Department of Mechanical Engineering, Colorado School of Mines, Golden, CO 80401, USA; kasrataghikhani@mymail.mines.edu (K.T.); jberger@mines.edu (J.R.B.); sricote@mines.edu (S.R.); hzhu@mines.edu (H.Z.); 2 HyET Hydrogen USA LLC, Sacramento, CA 95826, USA; alexis.dubois@hyethydrogen.com

**Keywords:** electrochemistry, transport-induced stress, ceramic-proton-conducting membranes, BZY20

## Abstract

This paper reports an extended Nernst–Planck computational model that couples charged-defect transport and stress in tubular electrochemical cell with a ceramic proton-conducting membrane. The model is particularly concerned with coupled chemo-mechanical behaviors, including how electrochemical phenomena affect internal stresses and vice versa. The computational model predicts transient and steady-state defect concentrations, fluxes, stresses within a thin ${{\mathrm{BaZr}}_{0.8}Y_{0.2}O}_{3-\delta }$ (BZY20) membrane. Depending on the polarization (i.e., imposed current density), the model predicts performance as a fuel cell or an electrolyzer. A sensitivity analysis reveals the importance of thermodynamic and transport properties, which are often not readily available.

## 1. Introduction

Materials such as doped barium zirconates (e.g., ${{\mathrm{BaZr}}_{1-x}Y_{x}O}_{3-\delta }$, BZY) are perovskites that have good proton conductivity at intermediate temperatures (T≳500 °C) [1] and chemical stability in environments containing H${}_{2}$O and CO${}_{2}$ [1,2,3,4]. Thus, these materials are suitable as proton-conducting membranes for applications that depend on hydrogen separations (e.g., fuel cells) [5,6]. Protonic ceramic membranes have also been employed to compress hydrogen up to 50 bar. Methane is reformed in the presence of steam on the Ni-BZY based ceramic-metal support, and the produced hydrogen is galvanically driven through the membrane and compressed on the other side of the membrane [7]. Although BZY materials are predominantly proton conductors in moist reducing atmospheres, they are mixed ionic-electronic conductors (MIEC) in oxidizing atmospheres with the presence of O-site polarons. As the temperature increases above 600 °C, the conduction through oxygen vacancies becomes not negligible. The present paper focuses on the chemo-mechanical behavior of ${{\mathrm{BaZr}}_{0.8}Y_{0.2}O}_{3-\delta }$, called BZY20.

The concentration variations associated with charged-defect transport within the membrane cause lattice-scale strain and volume deformation, often referred to as chemical expansion [8,9]. Because ceramic materials are brittle, defect-induced stresses can initiate distortions, cracks, and fracture. Moreover, as discussed in early works by Lee [10] and Larché and Cahn [11,12], local stress gradients also contribute to defect fluxes [13].

Atkinson et al. [14,15] analyzed the effects of defect-induced stress in solid-oxide fuel cells (SOFCs) and oxygen-ion-conducting membranes with alternative geometries and mechanical constraints. Euser et al. developed an extended Nernst–Planck–Poisson (NPP) model to evaluate stress in planar and radial oxygen-separation membranes [16,17,18]. Andersson et al. [19] and Han et al. [20] used X-ray diffraction to measure the chemical expansion of BZY for different dopant levels in moist and dry environments. Marrocchelli et al. [21] and Bishop et al. [22] reported detailed descriptions of chemical expansion in the perovskite materials. Dubois et al. [23] developed a coupled chemo-thermo-mechanical model using an extended Nernst-Planck formulation and evaluated the stresses associated with a protonic-ceramic fuel cell (PCFC) in a button-cell configuration. Ricote et al. [24] used high-temperature X-ray diffraction (HT-XRD) and thermogravimetric analysis (TGA) to study the effects of oxygen partial pressure on lattice parameters and oxygen non-stoichiometry in ${{\mathrm{BaZr}}_{0.9}{\mathrm{Dy}}_{0.1}O}_{3-\delta }$ (BZDy10).

**Figure 1:**
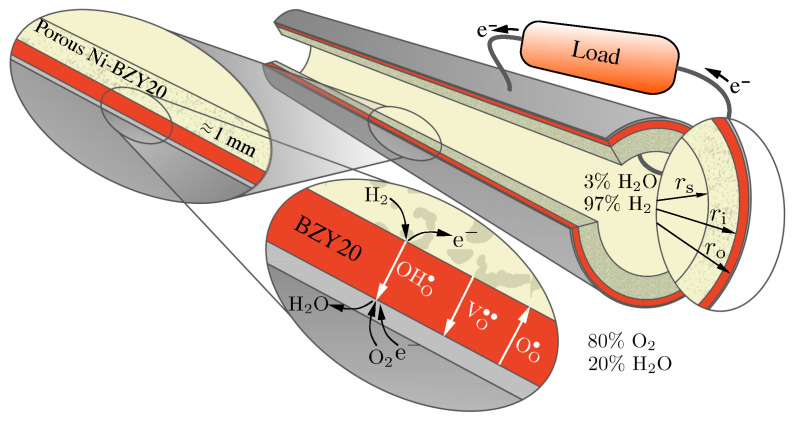
Proton-conducting tubular cell consisting of a dense BZY20 electrolyte supported by porous Ni-BZY20 operating as a fuel cell.

The current study develops an electro-chemo-mechanical computational model based on an extended Nernst–Planck (NP) formulation. The one-dimensional radial model considers a tubular configuration with a thin dense BZY20 membrane supported by a relatively thick porous composite electrode (Figure 1). The model predicts transient profiles of defect concentrations and stress states as functions of applied electrical current. As with all models, results depend on physical parameters, including thermodynamic and transport properties. A sensitivity analysis seeks to identify the most important parameters and properties.

## 2. Defect Chemistry

The structures and behaviors of proton-conducting ceramics have been documented in numerous reviews [1,2,19,25,26,27,28,29,30,31]. Oxides with ABO${}_{3}$ perovskite structures, such as ${\mathrm{BaZrO}}_{3}$, are among the most promising proton conductors. Effective proton conductivity is established by B-site doping with a trivalent cation such as yttrium (Y${}^{3+}$) to replace a fraction of the Zr${}^{4+}$, thus introducing oxygen vacancies to preserve local charge neutrality. The present study considers 20% Y doping, ${{\mathrm{BaZr}}_{0.8}Y_{0.2}O}_{3-\delta }$. The needed thermodynamic, transport, and mechanical properties are derived from prior publications [1,2,4,19].

Figure 1 illustrates aspects of the tubular geometry and the transport processes. The profiles and fluxes of three mobile charged defects (protons ${\mathrm{OH}}_{O}^{•}$, oxygen vacancies $V_{O}^{••}$ and polarons $O_{O}^{•}$) depend on the current density via an external circuit. Depending on the polarization (i.e., the direction of the electrical current), the cell may operate as a fuel cell or as an electrolyzer [32]. As illustrated in Figure 1, the present study considers gas-phase mixtures of 20% H_2_O and 80% O_2_ on the exterior electrode (positrode) and 97% H_2_ and 3% H_2_O on the interior electrode (negatrode).

Defect-incorporation reactions at the membrane boundaries may be stated in Kröger–Vink notation as

(1) $$\frac{1}{2}H_{2}+O_{O}^{•}⇌{\mathrm{OH}}_{O}^{•},$$

(2) $$\frac{1}{2}O_{2}+O_{O}^{\times }+V_{O}^{••}⇌2O_{O}^{•},$$

(3) $$H_{2}O+V_{O}^{••}+O_{O}^{\times }⇌2{\mathrm{OH}}_{O}^{•}.$$

Corresponding equilibrium constants for the incorporation reactions are

(4) $$K_{p,H_{2}}=\frac{{\left[{\mathrm{OH}}_{O}^{•}\right]}_{L}}{p_{H_{2}}^{1/2}{\left[O_{O}^{•}\right]}_{L}},$$

(5) $$K_{p,O_{2}}=\frac{{\left[O_{O}^{•}\right]}_{L}^{2}}{p_{O_{2}}^{1/2}{\left[O_{O}^{X}\right]}_{L}{\left[V_{O}^{••}\right]}_{L}},$$

(6) $$K_{p,H_{2}O}=\frac{{\left[{\mathrm{OH}}_{O}^{•}\right]}_{L}^{2}}{p_{H_{2}O}{\left[O_{O}^{\times }\right]}_{L}{\left[V_{O}^{••}\right]}_{L}},$$

where $p_{k}$ are the gas phase partial pressures and the defect concentrations are represented in lattice units. The defect molar concentrations may be evaluated using the molar volume (for BZY20, $V_{m}=4.57\times 10^{-5}$ m^3^ mol^−1^) as

(7) $${\left[X_{k}\right]}_{L}=\left[X_{k}\right]V_{m}.$$

The present model includes polaron traps [4]. In other words, some fraction of the otherwise highly mobile small polarons can be trapped, thus immobilized in the proximity of the yttrium dopant. Stated as a reaction,

(8) $$Y_{\mathrm{Zr}}^{\prime }+O_{O}^{•}⇌\left(Y_{\mathrm{Zr}}^{\prime }-O_{O}^{•}\right).$$

The associated equilibrium constant follows as

(9) $$K_{p,\mathrm{Trap}}=\frac{{\left[\left(Y_{\mathrm{Zr}}^{\prime }-O_{O}^{•}\right)\right]}_{L}}{{\left[Y_{\mathrm{Zr}}^{\prime }\right]}_{L}{\left[O_{O}^{•}\right]}_{L}}.$$

The present model also assumes that the gas phases are equilibrated on both sides of the cell,

(10) $$H_{2}+\frac{1}{2}O_{2}⇌H_{2}O.$$

Because the gas phase is equilibrated, the defect-incorporation equilibrium constants are not all independent; they are constrained as

(11) $$K_{p,H_{2}}^{2}K_{p,O_{2}}=K_{p,H_{2}O}K_{p,G}.$$

The equilibrium constants for each reaction can be evaluated via thermodynamic properties as

(12) $$K_{p}=\exp\left(-\frac{\Delta G^{\circ }}{RT}\right)=\exp\left(-\frac{\Delta H^{\circ }}{RT}\right)\exp\left(\frac{\Delta S^{\circ }}{R}\right),$$

where *T* is temperature, *R* is the gas constant, $\Delta G^{\circ }$ is the change in Gibbs free energy, $\Delta S^{\circ }$ is the change in defect entropy, and $\Delta H^{\circ }$ represents change in enthalpy. Table 1 lists the thermodynamic properties used in the present study [4].

Assuming the defect reactions at the membrane surfaces are at equilibrium, defect concentrations at surfaces are constants that depend on the gas phase composition. Evaluating the boundary concentrations must consider site and electroneutrality balances. Charge neutrality requires that

(13) $$2{\left[V_{O}^{••}\right]}_{L}+{\left[{\mathrm{OH}}_{O}^{•}\right]}_{L}+{\left[O_{O}^{•}\right]}_{L}-{\left[Y_{\mathrm{Zr}}^{\prime }\right]}_{L}=0.$$

The ABO_3_ pervoskite structure further constrains the oxygen site balance as

(14) $${\left[V_{O}^{••}\right]}_{L}+{\left[{\mathrm{OH}}_{O}^{•}\right]}_{L}+{\left[O_{O}^{•}\right]}_{L}+{\left[O_{O}^{x}\right]}_{L}+{\left[\left(Y_{\mathrm{Zr}}^{\prime }-O_{O}^{•}\right)\right]}_{L}=3.$$

The yttrium doping is fixed as ${\left[Y_{\mathrm{Zr}}^{\prime }\right]}_{L}^{\circ }=0.2$, which also constrains the trap balance as

(15) $${\left[Y_{\mathrm{Zr}}^{\prime }\right]}_{L}+{\left[\left(Y_{\mathrm{Zr}}^{\prime }-O_{O}^{•}\right)\right]}_{L}={\left[Y_{\mathrm{Zr}}^{\prime }\right]}_{L}^{\circ }.$$

The defect concentrations at the boundaries are calculated by solving Equations (5), (6), (9), (13)–(15) simultaneously. Algorithmic details about the calculation may be found in Zhu et al. [4].

## 3. Extended NP Membrane Model

The defect concentrations are governed by a conservation principle, represented mathematically as

(16) $$\frac{\partial [X_{k}]}{\partial t}+\nabla •J_{k}={\dot{\omega }}_{k},$$

where *t* is time, $J_{k}$ are defect transport fluxes, and ${\dot{\omega }}_{k}$ are the net molar production rates of the polaron traps within the membrane [4]. In the current work, standard Nernst–Planck fluxes are extended to incorporate the contribution from hydrostatic-stress gradients. The defect-transport fluxes include the effects of diffusion $J_{k}^{D}$, migration, $J_{k}^{M}$, and hydrostatic stress $J_{k}^{S}$,

(17) $$J_{k}=J_{k}^{D}+J_{k}^{M}+J_{k}^{S}.$$

The flux may be represented as

(18) $$J_{k}=-D_{k}\left(\nabla \left[X_{k}\right]+\frac{z_{k}F}{RT}\left[X_{k}\right]\nabla \Phi_{e}-\frac{3\beta_{k}}{RT}\left[X_{k}\right]\nabla \sigma_{h}\right),$$

where $D_{k}$ are defect diffusion coefficients, $z_{k}$ are the defect’s charge, $\Phi_{e}$ is the electrostatic potential, $\beta_{k}$ is the coefficient of chemical expansion, and $\sigma_{h}$ is the hydrostatic stress. The defect charges are $z_{V_{O}^{••}}=+2$, $z_{{\mathrm{OH}}_{O}^{•}}=+1$, and $z_{O_{O}^{•}}=+1$. Hydrostatic stress within the membrane is defined as

(19) $$\sigma_{h}=\frac{\sigma_{rr}+\sigma_{\theta \theta }+\sigma_{zz}}{3}.$$

The polaron traps are produced and consumed according the reaction represented by Equation (8). The rate of progress of the trap reaction may be evaluated via mass-action kinetics as

(20) $${\dot{q}}_{\mathrm{Trap}}=k_{f}\left[Y_{\mathrm{Zr}}^{\prime }\right]\left[O_{O}^{•}\right]-k_{b}\left[\left(Y_{\mathrm{Zr}}^{\prime }-O_{O}^{•}\right)\right],$$

where $k_{f}$ and $k_{b}$ are forward and backward rate constants, which are related through the equilibrium constant. The present model assumes $k_{f}=10^{6}$. The species production rate due to the trap reaction can be expressed as ${\dot{\omega }}_{Y_{\mathrm{Zr}}^{\prime }}=-{\dot{q}}_{\mathrm{Trap}}$, ${\dot{\omega }}_{O_{O}^{•}}=-{\dot{q}}_{\mathrm{Trap}}$, and ${\dot{\omega }}_{\left(Y_{\mathrm{Zr}}^{\prime }-O_{O}^{•}\right)}={\dot{q}}_{\mathrm{Trap}}$.

### 3.1. Chemical-Expansion Coefficient

The chemical-expansion coefficient $\beta_{k}$ is defined to be the change in lattice volume with respect to the changes in defect concentration. As reported by Andersson et al. [19], the present model uses $\beta_{{\mathrm{OH}}_{O}^{•},\;L}=0.033$ (in lattice units). In molar units, $\beta_{k}=\beta_{{\mathrm{OH}}_{O}^{•},\;L}V_{m}=1.5\times 10^{-6}$ m^3^ mol^−1^. Ricote et al. [24] showed that the O-site polarons produce negligible chemical expansion. The present model attributes all chemical expansion to the protons, neglecting lattice distortion due to polarons and oxygen vacancies.

### 3.2. Electrostatic Potential $\Phi_{e}$

The electrostatic potential may be calculated by solving the Gauss equation,

(21) $$\frac{\partial E}{\partial t}=\frac{1}{\varepsilon_{0}\varepsilon_{r}}\left[i-\sum_{k}z_{k}FJ_{k}\right],$$

where the electrostatic-potential field is $E=\nabla \Phi_{e}$, $\varepsilon_{0}$ and $\varepsilon_{r}$ are vacuum and relative permittivities, respectively, and *i* is the external current density. However, the present model implements an approximately equivalent method by enforcing strict local electroneutrality. In this limit, the local charge flux must vanish as

(22) $$\sum_{k}z_{k}F\left[X_{k}\right]=0.$$

The electrostatic potential on one of the boundaries is set to a reference $\Phi_{e}=0$, while the other boundary is set to impose the net current density *i* through the membrane. In other words, the charge flux through the membrane is exactly balanced by the current density *i* through the external circuit,

(23) $$\sum_{k}z_{k}FJ_{k}=i.$$

This algebraic equation, together with Equation (16), is sufficient to determine the local electrostatic-potential gradient $\nabla \Phi_{e}$.

### 3.3. Defect Diffusion Coefficients

The charged-defect diffusion coefficients are represented in an Arrhenius equation as

(24) $$D_{k}=D_{k}^{\circ }\exp\left(-\frac{E_{k}}{RT}\right),$$

where $D_{k}^{\circ }$ and $E_{k}$ are pre-exponential factors and activation energies, respectively. As reported by Zhu et al. [4], Table 2 lists the values of $D_{k}^{\circ }$ and $E_{k}$ for three mobile charged defect.

### 3.4. Stress, Strain, and Displacement

The isotropic stress–strain relationship including chemically induced stress may be written as

(25) $$\sigma =\lambda \;\mathrm{tr}\left(\epsilon \right)I+2G\epsilon -\left(3\lambda +2G\right)\left(\sum_{k}\beta_{k}\Delta \left[X_{k}\right]\right)I.$$

where σ is the stress tensor, ϵ is the strain tensor, *I* is the unit tensor, and $\Delta [X_{k}]$ represents the change in concentration with respect to a reference zero-strain state. The current study does not incorporate the residual strain related to the fabrication process [5,23]. The Lamé constant λ and shear modulus *G* are defined as

(26) $$\begin{array}{cc} \lambda = & \;\frac{E_{m}\nu_{m}}{\left(1+\nu_{m}\right)\left(1-2\nu_{m}\right)}, \end{array}$$

(27) $$\begin{array}{cc} G= & \;\;\frac{E_{m}}{2(1+\nu_{m})}. \end{array}$$

In these expressions, $E_{m}$ is the Young’s modulus, and $\nu_{m}$ is Poisson’s ratio of the membrane. The tube is assumed to be infinitely long, resulting in a plane-strain state. Consequently, the strain components in the axial *z* direction vanish,

(28) $$\epsilon_{zz}=\epsilon_{zr}=\epsilon_{z\theta }=0.$$

The strain–displacement relationships with respect to radial displacement *u* are expressed as

(29) $$\epsilon_{rr}=\frac{\partial u}{\partial r},\;\epsilon_{\theta \theta }=\frac{u}{r},\;\epsilon_{r\theta }=0.$$

Assuming a quasi-static stress field and the absence of body forces, the divergence of the stress must vanish,

(30) ∇•σ=0

In one-dimensional radial coordinates, the stresses can be represented as

(31) $$\frac{\partial \sigma_{rr}}{\partial r}+\frac{1}{r}\frac{\partial \sigma_{r\theta }}{\partial \theta }+\frac{\partial \sigma_{rz}}{\partial z}+\frac{\sigma_{rr}-\sigma_{\theta \theta }}{r}=0.$$

Considering the tube to be long and axisymmetric, variations in axial *z* and circumferential θ stresses can be neglected. The radial component of the stress equilibrium simplifies to

(32) $$\frac{\partial \sigma_{rr}}{\partial r}+\frac{\sigma_{rr}-\sigma_{\theta \theta }}{r}=0.$$

In practice, protonic-ceramic membranes should be as thin as possible to promote high proton fluxes (usually on the order of tens of microns). Such very thin membranes must be supported with relatively thick porous support structures that also serve as electrodes (cf., Figure 1). In reducing environments (e.g., anodes of protonic-ceramic fuel cells) the most widely used supports are composites of Ni-BZY. Such structures provide the mechanical strength and stability. The percolating Ni phase serves dual roles as the hydrogen-reduction catalyst and the electrical conductor. Table 3 lists the dimensions of tube used for the present study. The membrane is 10 μm thick and the porous support is 1 mm thick. The outer electrode is specified to be thin, but not explicitly included in the present model.

The current study, which specifically focuses on the electro-chemo-mechanical behavior within the membrane, neglects defect transport and chemical expansion within the porous support. Table 3 also includes mechanical properties of BZY20 and Ni-BZY20. These properties were determined experimentally using the ultrasonic measurements [23].

The mechanical aspects of the present model follow the approach reported by Euser et al. [17]. By substituting the stress–strain relationships (Equation (25)) and strain–displacement relationships (Equation (29)), the equilibrium equation (Equation (32)) can be solved analytically to produce the local displacements *u*, assuming a perfect bond between support and membrane properly satisfies continuity of displacement and traction at the membrane boundary. The constants of integration can be evaluated assuming inner and outer surfaces of the tube are traction-free. Using these displacements, the local stress components within the membrane and porous support can be evaluated.

### 3.5. Computational Implementation

The computational model is formulated using the method-of-lines and a one-dimensional radial finite-volume mesh network. The model incorporates two-way coupling between the defect transport and stress in the membrane. Transport induced stress is evaluated using the analytical solution. Euser et al. [17] reported the radial displacement and stress for the tubular membrane, respectively, as

(33) $$u\left(r\right)=\frac{1+v_{m}}{1-v_{m}}\frac{I(r)}{r}+C_{1}r+\frac{C_{2}}{r},$$

(34) $$\begin{array}{cc} \sigma_{rr}\left(r\right)= & -\frac{E_{m}}{\left(1-v_{m}\right)}\frac{I(r)}{r^{2}}+\frac{E_{m}}{\left(1+v_{m}\right)\left(1-2v_{m}\right)}C_{1}\\ \; & -\frac{E_{m}}{\left(1+v_{m}\right)}\frac{C_{2}}{r^{2}}, \end{array}$$

where $C_{1}$ and $C_{2}$ are constants of integration, which can be calculated using the mechanical boundary conditions, as discussed earlier. In addition,

(35) $$I\left(r\right)=\int_{r_{i}}^{r}r\sum_{k}\beta_{k}\Delta \left[X_{k}\right]dr.$$

where $r_{i}$ is the inner radius of membrane. The transport problem is solved numerically. Equation (16) is effectively a parabolic partial differential equation that is solved in Matlab using the ode15i function. The second-order flux derivatives are approximated using conservative central differences. The migration $J_{k}^{M}$ and stress $J_{k}^{M}$ fluxes are discretized using an up-winding scheme, where the coefficient of $\left[X_{k}\right]$ behaves as an artificial velocity [33,34]. The simulations run efficiently and quickly on a typical personal computer.

## 4. Results and Discussion

### 4.1. Concentration Profiles

**Figure 2:**
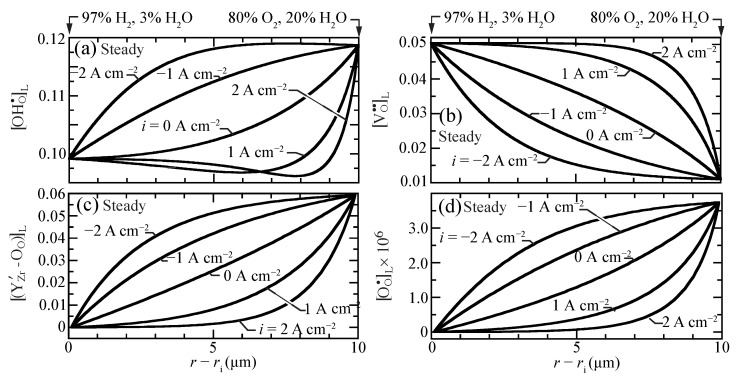
Charged-defect concentration profiles (lattice units) across the membrane for imposed current densities (i=−2,−1,0,1,2 A cm${}^{-2}$) at 600 ${}^{\circ }$C. (**a**) protons, (**b**) oxygen vacancies, (**c**) trapped polarons, and (**d**) O-site polarons.

Figure 2 shows the predicted steady-state lattice-scale concentration profiles ${\left[X\right]}_{L}$ of charged defects for current densities ranging between i=−2 A cm${}^{-2}$ (electrolysis) and i=2 A cm${}^{-2}$ (fuel cell). The proton concentrations are larger than, but comparable to, the vacancy and the trap concentrations. The O-site polaron concentrations are much lower. Even at open circuit, there is significant curvature in the defect-concentration profiles. The sense of the curvatures changes depending on the polarization (i.e., direction of the charge flux). Under fuel-cell polarization (i>0), the profiles reveal boundary-layer behavior near the O${}_{2}$/H${}_{2}$O side of the membrane. Interestingly, under very strong fuel-cell polarization, a local minimum appears in the proton profile.

**Figure 3:**
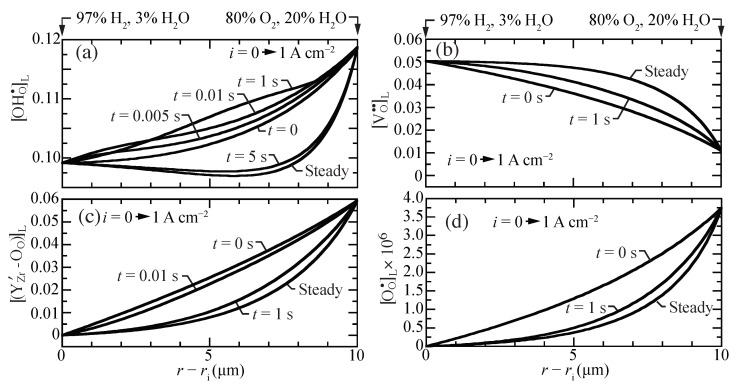
Charged-defect concentration profiles (lattice units) during a transient between open circuit and a suddenly imposed current density of i=1 A cm${}^{-2}$. The cell temperatures is fixed at 600 ${}^{\circ }$C. (**a**) protons, (**b**) oxygen vacancies, (**c**) trapped polarons, and (**d**) O-site polarons.

Figure 3 illustrates transient behavior of the concentration profiles, beginning from open-circuit conditions as a current density of i=1 A cm${}^{-2}$ is suddenly imposed. The characteristic time constant for the transient is on the order of one second. The vacancies, traps, and polarons behave as may be anticipated, transitioning smoothly between the two polarization states. The proton concentrations reveal somewhat more unusual behavior. The proton concentrations initially increase, before later approaching the lower concentrations at the i=1 A cm${}^{-2}$ steady-state. Although the magnitudes of the concentration variations are not large, there could be implications on the local transient stresses.

### 4.2. Proton Flux Profiles

**Figure 4:**
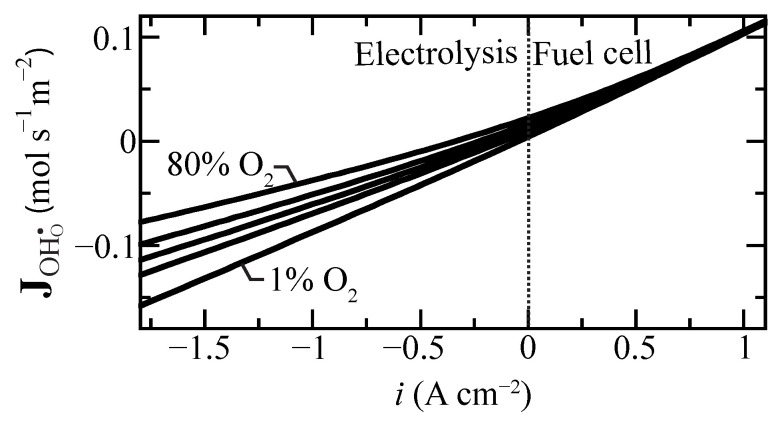
Protonic flux change as a function of current density for selected air side oxygen compositions ranging from 1% to 80% at 600 ${}^{\circ }$C. The oxygen concentration is balanced with steam at the boundary.

Proton flux is an important measure of membrane performance. Figure 4 shows the predicted proton flux as a function of current density for selected oxygen concentrations at the tube’s exterior electrode. Interestingly, under fuel-cell polarization (i>0), the flux is only weakly affected by the O${}_{2}$ concentration. The effect is much more significant under electrolysis polarization. As may be anticipated, high steam concentrations (i.e., low O${}_{2}$ concentration) lead to higher proton fluxes under electrolysis conditions.

**Figure 5:**
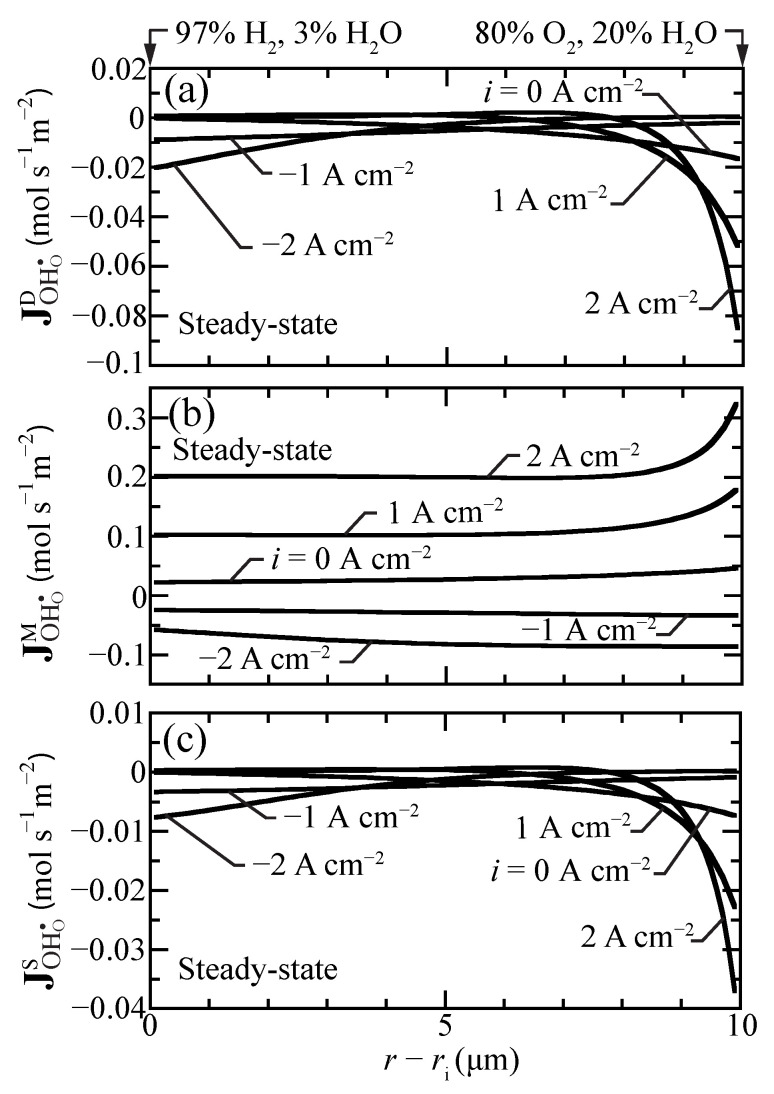
Steady-state proton-flux contributions for selected current densities (i=−2,−1,0,1,2 A cm${}^{-2}$) at 600 ${}^{\circ }$C. (**a**) diffusion flux, (**b**) migration flux, and (**c**) stress-induced flux.

At steady state, the defect-flux profiles must be essentially flat (i.e., very little spatial dependence). However, because of the radial coordinates, there will be very slight radial variations in the steady-state fluxes. The net proton flux comprises diffusion, migration, and stress contributions (Equation (17)). These flux contributions do vary spatially, even at steady state. Figure 5 plots separately the individual steady-state flux-contribution profiles under a range of imposed current densities. These results show that the migration contributions generally dominate. Especially under fuel-cell polarization, the diffusion and stress contributions have strong gradients near the outer boundary. The behaviors are consistent with those reported by Euser et al. [16,17] for La${}_{0.6}$Sr${}_{0.4}$Co${}_{0.8}$Fe${}_{0.2}$O${}_{3-\delta }$ (LSCF) oxygen-transport membranes.

**Figure 6:**
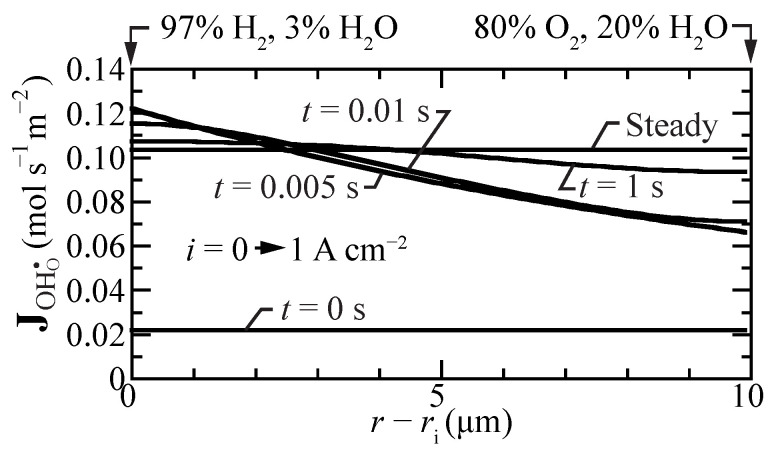
Transient proton-flux profiles within the membrane, beginning at open circuit mode and suddenly imposing a fuel-cell current density i=1 A cm${}^{-2}$. The cell temperature is fixed at 600 ${}^{\circ }$C.

Figure 6 shows proton-flux profiles during a transient between open circuit and a suddenly imposed current density of i=1 A cm${}^{-2}$. Even under open-circuit conditions, there is a small proton flux. Upon polarization, the flux profiles have spatial gradients, with the highest fluxes near the hydrogen fuel boundary. However, within a few seconds, the proton-flux profile becomes spatially uniform again at a significantly higher value, driving protons from the fuel side toward the oxygen side.

### 4.3. Stress Profiles

**Figure 7:**
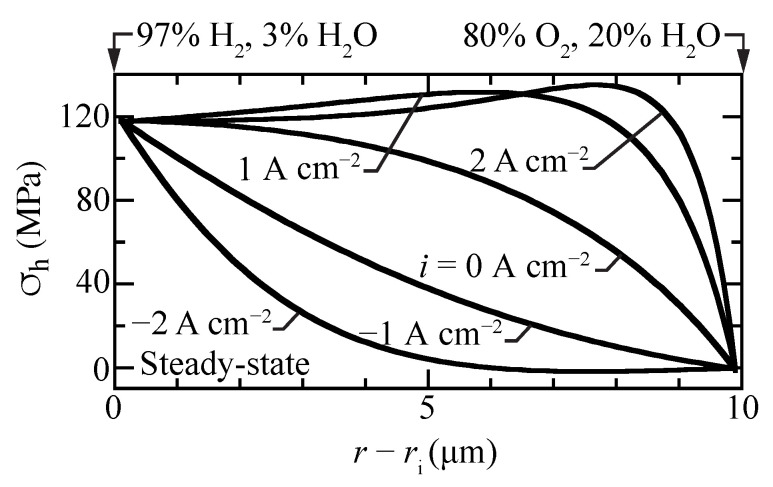
Steady-state hydrostatic stress profiles for selected current densities with the cell operating at 600 ${}^{\circ }$C.

Figure 7 shows hydrostatic stress profiles within the membrane for different imposed current densities. The stresses in the fuel cell mode (i>0) are considerably higher than in electrolysis mode (i<0). The fuel-cell stresses have local maxima within the membrane, slightly exceeding the high fuel-side boundary stresses. Moreover, the fuel-cell stress gradients are high, especially near the oxygen boundary. The curvature of the hydrostatic-stress profiles is positive for electrolysis, while it is negative at open circuit and in fuel-cell operation. Under strong electrolysis polarization, there can be a slight local minimum in the steady-state hydrostatic-stress profile. The present model attributes chemical strain to the protons. Comparing Figure 2a and Figure 7 shows that the correlation between the proton concentration profile and the hydrostatic stress profiles is evident.

**Figure 8:**
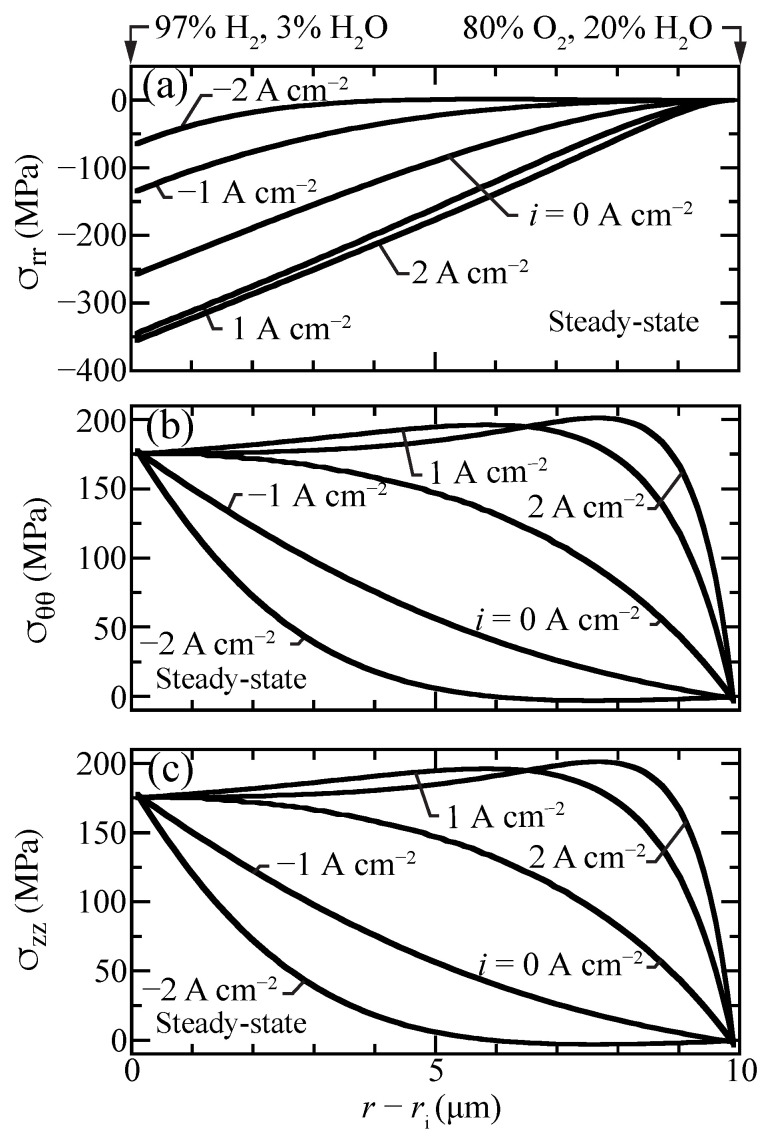
Steady-state stress-contribution profiles for selected current densities (i=−2,−1,0,1,2 A cm${}^{-2}$) with the cell operating at 600 ${}^{\circ }$C. (**a**) radial, (**b**) hoop, and (**c**) axial stress components.

Figure 8 shows profiles of the normal stress components that contribute to the hydrostatic stress. The radial stresses are compressive while hoop and axial stresses are tensile and nearly identical. The hydrostatic stress distribution is similar to hoop and axial stresses, although with lower magnitude since radial stresses are compressive (Figure 7). The stresses in the porous support are negligible, as chemical expansion in the support is omitted.

**Figure 9:**
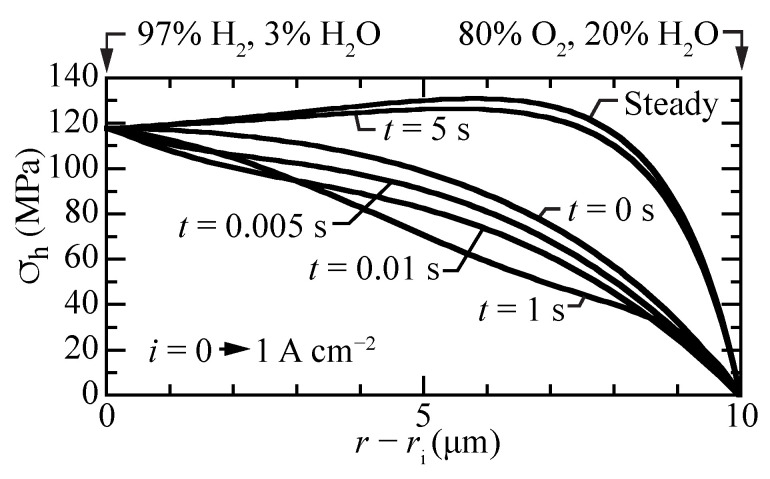
Transient hydrostatic stress profiles within the membrane, beginning at open circuit and suddenly imposing a fuel-cell current density of i=1 A cm${}^{-2}$.

Figure 9 shows the transient response of the hydrostatic stress as a current density of i=1 A cm${}^{-2}$ is suddenly imposed from an initial open-circuit steady-state condition. The local stresses decrease at early times, with local upward curvature at a time of around one second. By about five seconds, the steady-state hydrostatic-stress profile is achieved. The transient stress profiles behave similar to the transient proton concentrations profiles (Figure 3a).

**Figure 10:**
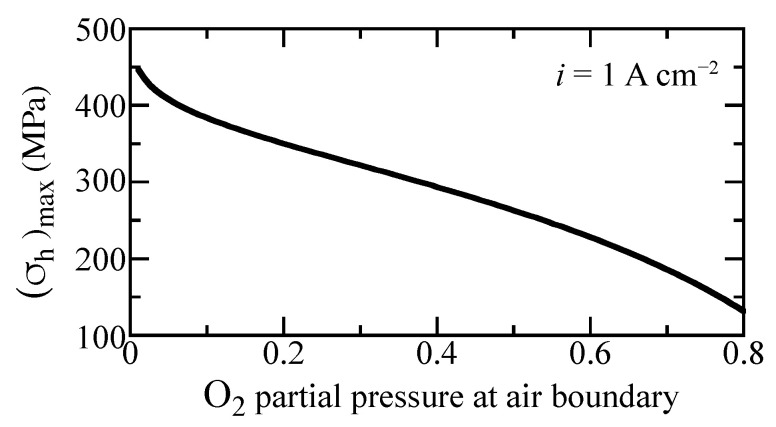
Maximum hydrostatic stress as a function of oxygen fraction at the outer boundary under i=1 A cm${}^{-2}$ imposed current density. The oxygen fraction is balanced with steam. The cell is fixed at a temperature of 600 ${}^{\circ }$C.

In a practically operating cell, the gas compositions and temperature can vary owing to reagent depletion, dilution, etc. Cell electrochemical performance generally depends on gas-phase concentrations. Local stresses are also affected by gas-phase composition. Figure 10 shows the maximum hydrostatic stress as a function of oxygen concentration for the imposed current density of i=1 A cm${}^{-2}$. As the oxygen partial pressure at the cathode boundary increases, the maximum hydrostatic stress decreases considerably. This curve does not depend significantly on the current density. Consequently, maintaining high oxygen concentration could enhance mechanical integrity.

### 4.4. Sensitivity Analysis

**Figure 11:**
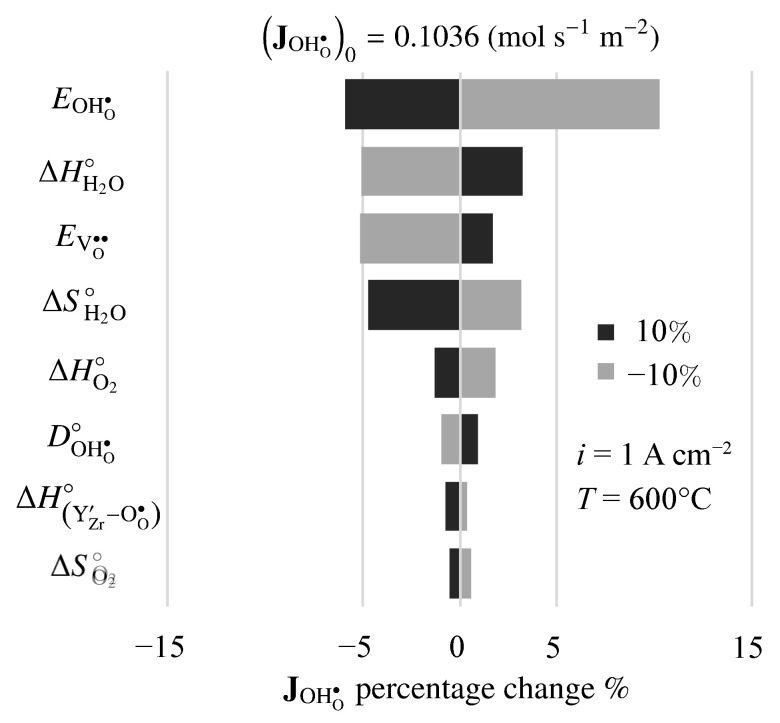
Sensitivity of steady-state fuel-cell proton flux to 10% changes in electro-chemo-mechanical properties. The imposed current density is i=1 A cm${}^{-2}$.

There is considerable uncertainty in some of the physical properties for BZY20. Thus, it is interesting to investigate the sensitivity of predicted results to the model’s properties and parameters. Figure 11 is a tornado plot that shows the effects of varying individual properties by ±10% on the predicted proton flux under fuel-cell polarization. The nominal model-predicted proton flux is $J_{{\mathrm{OH}}_{O}^{•}}=0.1036$ mol s${}^{-1}$ m${}^{-2}$. Under these conditions, the highest sensitivities were found to be the activation energy of the proton diffusion coefficient $E_{{\mathrm{OH}}_{O}^{•}}$, the enthalpy of the steam-incorporation reaction $\Delta H_{H_{2}O}^{\circ }$, the activation energy of the vacancy diffusion coefficient $E_{V_{O}^{••}}$, and the entropy of the steam-incorporation reaction $\Delta S_{H_{2}O}^{\circ }$.

**Figure 12:**
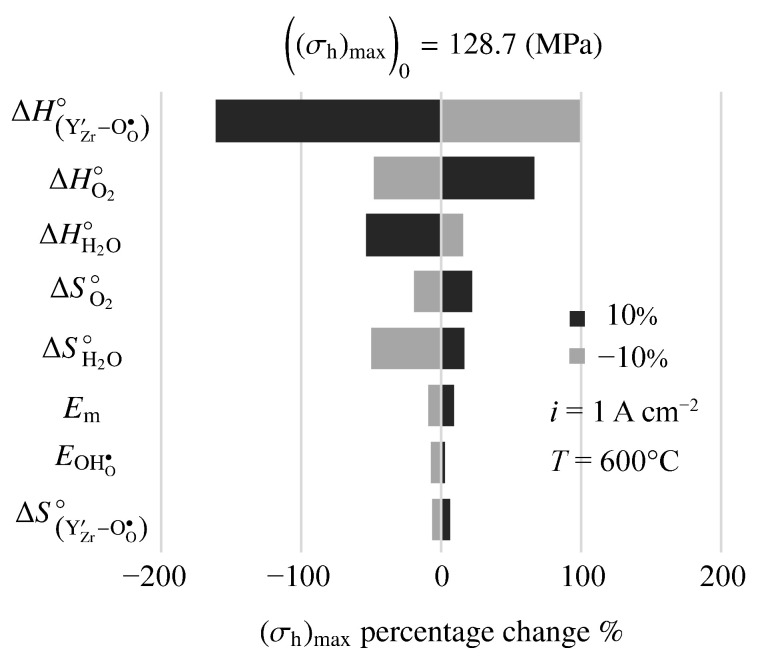
Sensitivity of maximum hydrostatic stress to 10% changes in electro-chemo-mechanical properties. The imposed current density is i=1 A cm${}^{-2}$.

Figure 12 is a tornado plot showing sensitivities of model-predicted maximum hydrostatic stress to model parameters. The cell is operating at 600 ${}^{\circ }$C with an imposed fuel-cell current density of i=1 A cm${}^{-2}$. Under these conditions, the nominal maximum hydrostatic stress is $\sigma_{h}=128.7$ MPa. The highest sensitivities are to the thermodynamic properties (enthalpy $\Delta H^{\circ }$ and entropy $\Delta S^{\circ }$) of the defect-incorporation reactions. Interestingly, mechanical properties (e.g., Young’s modulus, $E_{m}$) show relatively small sensitivity.

The present model uses best-available properties [4]. However, results of the sensitivity analysis point to needs for measuring, improving, and validating thermodynamic and transport properties. Such research is ongoing, but is a long-term process.

## 5. Summary and Conclusions

The performance of BZY20 as a proton-conducting fuel-cell or electrolysis membrane is simulated using an extended Nernst–Planck model. The model considers the one-dimensional radial behavior in a long tubular cell. The Nernst–Planck fluxes depend on local gradients of defect concentration, electrostatic potential, and hydrostatic stress. The model predicts transient and steady state profiles of defect concentrations and stress within a thin (10 μm) BZY20 membrane that is supported on a relatively thick porous Ni/BZY20 composite electrode. The present model concentrates on defect transport within the dense membrane, assuming ideal electrodes. In other words, charge-transfer polarization at the electrodes is neglected. The three charged defects (protons, oxygen vacancies, and small polarons) are incorporated into the membrane via equilibrated defect-incorporation reactions.

An important measure of membrane performance is the proton flux. However, as a practical matter, internal stresses are also important because of potential damage mechanisms with brittle ceramic materials. The present model couples the electrochemical performance and chemo-mechanical performance. A sensitivity analysis reveals an ongoing need to validate material-specific thermodynamic, transport, and mechanical properties, thus improving predictive capabilities of the models.

## Figures and Tables

**Table 1:** Enthalpy and entropy of defect incorporation reactions in BZY20. Reproduced with permission from Zhu et al. [4]. Copyright The Electrochemical Society, 2018.

Reaction	$\Delta H^{\circ }$ $\left(\mathrm{kJ}\;{\mathrm{mol}}^{-1}\right)$	$\Delta S^{\circ }$ $(J\;{\mathrm{mol}}^{-1}\;K^{-1})$
$\frac{1}{2}H_{2}+O_{O}^{•}⇌{\mathrm{OH}}_{O}^{•}$	−228.36	−54.80
$\frac{1}{2}O_{2}+O_{O}^{\times }+V_{O}^{•}⇌2O_{O}^{•}$	115.31	−45.89
$H_{2}O+V_{O}^{•}+O_{O}^{\times }⇌2{\mathrm{OH}}_{O}^{•}$	−93.30	−100.00
$Y_{\mathrm{Zr}}^{\prime }+O_{O}^{•}⇌\left(Y_{\mathrm{Zr}}^{\prime }-O_{O}^{•}\right)$	−90.30	−6.71
$H_{2}+\frac{1}{2}O_{2}⇌H_{2}O$	−248.11	−55.48

**Table 2:** Pre-exponential factors and activation energies for diffusivities of mobile charged defects in BZY20. Reproduced with permission from Zhu et al. [4]. Copyright The Electrochemical Society, 2018.

Charged Defects	$D_{k}^{\circ }$ (m${}^{2}$ s${}^{-1}$)	$E_{k}$ (kJ mol${}^{-1}$)
OH${}_{O}^{•}$	5.18 $\times 10^{-7}$	60.66
V${}_{O}^{••}$	2.03 $\times 10^{-7}$	85.19
O${}_{O}^{•}$	1.38 $\times 10^{-5}$	7.18

**Table 3:** Thermo-mechanical and geometrical properties of model.

Parameter	Value
Temperature, *T*	$600{\;}^{\circ }$C
Molar volume, $V_{m}$	$4.57\times 10^{-5}\;m^{3}\;{\mathrm{mol}}^{-1}$ [4]
Outer support radius, $r_{s}$	$4\times 10^{-3}$ m
Inner membrane radius, $r_{i}$	$5\times 10^{-3}$ m
Outer membrane radius, $r_{o}$	$5.01\times 10^{-3}$ m
Membrane Young’s modulus, $E_{m}$	205 GPa
BZY20	
Support Young’s modulus, $E_{s}$	108 GPa
Ni-BZY20 (Dry–25% porosity)	
Membrane Poisson’s ratio, $\nu_{m}$	0.24
BZY20	
Support Poisson’s ratio, $\nu_{s}$	0.27
Ni-BZY20 (Dry–25% porosity)	
